# Reply to ‘Hypothermic machine perfusion before viability testing of previously discarded human livers’

**DOI:** 10.1038/s41467-021-21183-7

**Published:** 2021-02-12

**Authors:** Hynek Mergental, Richard W. Laing, Simon C. Afford, Darius F. Mirza

**Affiliations:** 1 Liver Unit, Queen Elizabeth Hospital, University Hospitals Birmingham NHS Foundation Trust, Birmingham, UK; 2 National Institute for Health Research (NIHR), Birmingham Biomedical Research Centre, University of Birmingham and University Hospitals Birmingham NHS Foundation Trust, Birmingham, UK; 3 Centre for Liver and Gastrointestinal Research, Institute of Immunology and Immunotherapy, University of Birmingham, Birmingham, UK

**Keywords:** Diagnostic markers, Liver diseases, Hepatology

**Replying to** Otto B. van Leeuwen et al. *Nature Communications* 10.1038/s41467-021-21182-8 (2021)

We read with interest the comments regarding our VITTAL study report, submitted to *Nature Communications* by Dr. van Leeuwen and colleagues^1^, and we would like to take the opportunity to clarify the issues they highlighted regarding our trial novelty, the tested viability criteria, and use of the end-ischaemic normothermic perfusion alone^2^.

Firstly, we want to thank the authors for their compliments acknowledging the challenges of performing complex, high-risk liver transplant trials, as this context is important to the interpretation of the results of such research. Machine perfusion is a rapidly developing field that changes many aspects of transplantation medicine, and in the particular context of normothermic liver perfusion (NMP), minimises ischaemia reperfusion injury, allows significantly longer organ preservation, enables liver viability testing, and provides opportunity for therapeutic interventions^3–6^. The assessment of liver viability was pioneered by several groups, who independently developed different protocols for assessing transplantability, taking the risks of exploring the boundaries of liver utilisation in the era of closely scrutinised programmes’ outcomes^7–9^. While the potential benefits of NMP compared to static cold storage are many, the added cost and complexity of the procedure means that the transplant community looks for compelling clinical evidence to justify its use^10^.

Our group completed the proof-of-concept transplant series with discarded livers in 2015, and immediately proceeded to further rigorous testing with updated viability criteria within a clinical trial framework^7,11^. The VITTAL study linked primary endpoints of liver recovery rate and the 90-day graft survival. Worldwide, the risks of dying on the waiting list are significantly higher than the mortality after transplantation, and the organ utilisation parameters were used as proxy measures for access to the transplant treatment^12,13^. The inclusion of suboptimal livers in machine perfusion trials has been widely accepted by the transplant community, as these organs benefit most from the use of this novel technology^14,15^. Conducting studies with discarded livers in this context brings the greatest benefit to the patients from the local waiting list perspective, although some critics may view this particular inclusion criterion as being vague or subjective.

The motives for donor livers not being used are usually multifactorial, but can be grouped according to reasons relating to the donor, the recipient or logistics. VITTAL aimed to explore the limits of the highest-risk donor liver utilisation and intended to include livers discarded due to donor-related factors only, to allow an unambiguous reproducibility of its findings. We addressed the major challenge of achieving this objective by using a two-tier process of liver inclusion. However, the study design also needed to ensure recipient safety and address the ethical dilemma of not including discarded livers of better quality than required for trial inclusion (which would very likely meet the viability criteria and lead to superior post-transplant results), as outlined in the manuscript Supplementary material.

The time lag between pilot clinical findings and their validation by a prospective trial is the major pitfall of producing high-level evidence in this rapidly evolving field. This means that today’s discovery may take an average of 4–5 years to be published in a peer-reviewed journal, and this can take significantly longer if the project were funded by an independent body or required a multi-centre design^3,14,16^. The issue raised by Dr. van Leeuwen and colleagues regarding the use of viability criteria without considering bile production and composition is valid, and if we were designing the trial today we would have included those. VITTAL was designed, however, in 2015, and the patients enroled in the 16-month period from November 2016 to February 2018^11^, when the significance of bile composition for the development of non-anastomotic biliary strictures (NAS) in livers donated after circulatory death (DCD) was not yet known^8,9,17^. While DCD donors may represent the pool of organs with the greatest potential for future expansion, on the global scale this remains a niche area as >90% of liver transplants in the Western world are currently performed with livers donated after brain death (DBD)^12,15,18^. As a result, a large majority of livers are underutilised due to steatosis or logistical reasons (e.g., suboptimal livers that might be usable, but the excessive projected cold ischaemic time precludes their use). Considering the exceedingly low incidence of non-anastomotic biliary strictures in DBD livers, the testing of viability focused on hepatocellular function might be a preferable option^19^.

Adoption and implementation of machine perfusion into the liver transplantation pathway might have several indications, e.g., to avoid the devastating consequences of liver primary non-function and severe early dysfunction. The end-ischaemic NMP viability assessment used in VITTAL was intended as a diagnostic tool to select transplantable livers from the pool of the highest-risk donors. The study demonstrated that 7 out of 10 livers can be safely transplanted with 100% 90-day graft survival. When we applied the Groningen viability criteria to the VITTAL cohort of 31 livers discarded by all UK transplant centres that met the pre-defined high-risk criteria, only one liver was deemed to be transplantable. This organ remained functioning at 24-months follow up, and while this proves that the criteria have high specificity to predict NAS, the sensitivity to predict graft loss within 90-days appears to be relatively low. Robust modelling would be required to quantify the net benefits of the improved access to transplantation on waiting list mortality, after deduction of the added patients needing re-transplantation for non-anastomotic biliary strictures. The 100% actuarial post-transplant patient survival in both the authors’ and our own series, however, suggests a clear benefit to receiving a transplant compared to remaining on the waiting list^2,9^. More research is needed to establish whether DBD and DCD livers should be assessed using the same or different criteria, and this is an ongoing interest of ours.

The incidence of NAS in DCD livers enrolled in the trial was nevertheless higher than anticipated. VITTAL was not powered to evaluate post-transplant biliary complications, and the only specific inclusion criterion for DCD livers was excessive donor warm ischaemia (defined as the period between the systolic blood pressure <50 mmHg to aortic perfusion >30 min). We were transparent in reporting the study outcomes, including the results of per-protocol performed magnetic resonance cholangiopancreatography scans. Our key messages are that end-ischaemic NMP did not cause biliary problems even in very marginal DBD livers, and that this type of perfusion did not appear to prevent development of non-anastomotic biliary strictures in high-risk DCD grafts; of the lost livers, one graft was exposed to 40 min of donor warm ischaemia, and the other two came from 69-year-old donors.

The Groningen group has a long track record in research on biliary complications in DCD transplants, and we expect their ongoing randomised trial will provide important evidence on the choice of optimal strategies to prevent NAS^17,20,21^. We eagerly await the results, as most of the studies published to date kept the donor warm ischaemia within the 30-min safety zone^9,22,23^. We appreciate the important technical remark regarding the benefits of using small diameter biliary catheters and the importance of avoiding technical issues, leading to a presumed lack of bile production^24,25^.

To conclude, the VITTAL trial demonstrated that objective assessment of high-risk donor livers can allow safe utilisation of up to 70% of currently discarded organs while achieving 100% 90-day graft survival. The study design introduced for the first time rigorous and reproducible high-risk inclusion criteria. While our viability measures achieved high-liver recovery rates, the addition of bile assessment might improve diagnostic accuracy regarding non-anastomotic biliary strictures in DCD livers.
